# Adult-Acquired Esotropia: Clinical Characteristics, Risk Factors and Outcomes of a Novel Surgical Approach

**DOI:** 10.3390/jcm15020747

**Published:** 2026-01-16

**Authors:** Diego José Torres García, Beatriz Pérez Morenilla, Ana Álvarez Gómez, Timoteo González-Cruces, Vanesa Díaz-Mesa, David Cerdán Palacios, Ana Morales Becerra

**Affiliations:** 1 Strabismus and Pediatric Ophthalmology Unit, Arruzafa Hospital, 14012 Córdoba, Spain; 2 Optometry Unit, Arruzafa Hospital, 14012 Córdoba, Spain; 3 Research, Development and Innovation Unit, Arruzafa Hospital, 14012 Córdoba, Spain

**Keywords:** acquired esotropia, adult strabismus, lateral rectus resection

## Abstract

**Objective:** We aimed to study acquired esotropia in adults and its risk factors, compile treatments performed and describe surgical technique used, with a novel indication. **Methods:** We conducted a retrospective study of patients with insidious distant esotropia along with distant horizontal diplopia (angles 2–30 PD with wide fusion amplitude): Refractively emmetropic, moderately myopic and mildly hyperopic. No systemic alterations. **Results:** 30 cases were included, average age: 38.13 ± 14.95. Mean time elapsed from the onset of symptoms to surgical treatment was 22.52. Mean spherical equivalent is −3.19 ± 2.83. Mean preoperative horizontal deviation was 18.58 ± 5.45 PD in distant vision and 5.48 ± 8.35 PD in close vision (*p* < 0.001). 100% of cases reported diplopia in distance vision. 20% required prismatic treatment (<10 PD) and 80% surgical (>10 PD) by lateral rectus resection, with an average of 4.82 ± 1.23 mm. Sensory result was successful in 100% of the cases and motor in 75%. **Conclusions:** We are facing a new type of acquired esotropia in adults that can be individualized by its clinical and therapeutic characteristics. Our prismatic and surgical treatment has been successful.

## 1. Introduction

Acquired adult esotropia is a rare form of strabismus, with an estimated prevalence of approximately 0.3%. Its classification is challenging due to the presence of multiple subgroups, each characterized by distinct etiopathogenic mechanisms. The condition typically manifests in late childhood, adolescence, or adulthood [1], while maintaining good binocular function. It represents an acquired form of esotropia in which the deviation at distance and near fixation is similar [1,2], with a difference of less than 5 prism diopters (PD) across various gaze positions. Patients usually present with constant or intermittent diplopia that progressively worsens. In acute cases with sudden onset, neuroimaging—particularly magnetic resonance imaging (MRI)—and a complete neurological assessment are generally warranted.

Cases categorized as non-organic are typically attributed to decompensated endophorias. Classically, three main subtypes have been described [3]:

- Type I (Swan type): Acute esotropia occurring after occlusion of one eye or disruption of binocular vision, possibly associated with underlying heterophoria (endophoria) and emmetropia. Management is exclusively surgical.
- Type II (Burian–Franceschetti type): Acute-onset esotropia without apparent precipitating factors, often linked to psychological or physical stress or trauma. Although neurological causes must always be excluded, treatment remains surgical.
- Type III (Bielschowsky type): Esotropia initially manifesting at distance fixation and later at near, typically associated with moderate uncorrected myopia, persistent accommodative convergence, and weak tonic divergence. The latter mechanism, refuted by Jampolsky, has also been hypothesized to result from bilateral abducens (cranial nerve VI) paresis.

More recently, several authors [3,4,5,6] have expanded the etiological classification of this disorder:

- Type IV, related to excessive use of mobile or digital devices [7,8].
- Type V (refractive–accommodative type), associated with high hyperopia, often manageable through optical correction alone.
- Type VI, characterized by microstrabismus, decompensated phorias, or, less frequently, by intracranial abnormalities such as brain tumors, Arnold–Chiari malformation, posterior fossa lesions, intracranial hypertension, or cerebellar ataxia. Neuroimaging and neurological evaluation are recommended for patients in this category.

Treatment strategies for acquired adult esotropia vary among authors [9,10]. Management approaches range from conservative measures, such as the use of prisms [10,11,12], to interventional techniques including bilateral botulinum toxin injections into the medial rectus muscles or extraocular muscle (EOM) surgery [9,10,13].

Despite the existing classifications and proposed etiological subtypes, the current literature does not fully encompass all clinical presentations of acquired adult esotropia encountered in contemporary clinical practice. Technological changes in modern lifestyles—particularly the widespread and prolonged use of digital devices—may be influencing the stability of the visual–motor system, contributing to an increasing incidence of strabismic disorders associated with diplopia. In this context, a subgroup of adult patients with acquired esotropia has been identified whose clinical profile does not fully conform to existing classifications, suggesting the presence of a previously underrecognized entity.

Accordingly, the present study aims to describe the common clinical characteristics of this cohort of patients with adult-acquired esotropia and to analyze their management, with particular emphasis on surgical treatment. Furthermore, the surgical approach and outcomes are contrasted with those reported in the current literature, in order to contribute to a better understanding of this emerging clinical group and to support an updated perspective on the classification and therapeutic strategies for adult-acquired esotropia

## 2. Materials and Methods

A retrospective study was conducted on data from patients diagnosed and treated for adult-acquired esotropia at Hospital Arruzafa (Córdoba, Spain). The study was approved by the hospital’s Ethics Committee, and data collection was performed in accordance with the principles of the Declaration of Helsinki.

### 2.1. Inclusion and Exclusion Criteria

The inclusion criteria comprised all adult patients diagnosed with and surgically treated for acquired esotropia between January 2017 and December 2023 who presented with diplopia. Eligible cases exhibited an insidious onset of transient episodes of horizontal diplopia at distance fixation, typically occurring when patients were fatigued—most frequently at night, while driving, or watching television. No diplopia was reported at near fixation, although a small-angle deviation, generally smaller than that at distance, and high convergence fusional amplitudes were often present (fusional vergence was assessed using horizontal prism bars, showing reduced divergence (<12 PD) and increased convergence (>30 PD).

Concretely, the study population was characterized by distance-predominant esotropia, defined as an esodeviation at distance fixation ranging from 6 to 25 prism diopters (PD), consistently associated with diplopia at distance vision. At near fixation, esodeviation ranged from 0 to 12 PD and was not associated with diplopia.

Exclusion criteria included any history of strabismus, ocular pathology, previous ocular surgery, and/or neurological disease.

### 2.2. Data Collection and Ophthalmological Examination

Preoperative data were extracted from medical records, including the patient’s age at first presentation and demographic information (sex, race, and age). Additional information was collected regarding the average number of daily hours spent using electronic devices, as reported by patients or, in the case of younger individuals, by family members. The following comprehensive ophthalmological examination was performed:

1. Refraction: Objective refraction under cycloplegia, obtained by instilling three drops of 1% cyclopentolate at 5-min intervals, followed by retinoscopy 30 min after the final instillation. The refractive status was expressed as the spherical equivalent, calculated by adding the spherical component to half the algebraic value of the cylindrical component. Myopia was classified as low (≤−3.00 D), moderate (−3.00 D to −6.00 D), or high (>−6.00 D).
2. Best-Corrected Visual Acuity (BCVA): Assessed for both distance and near vision.
3. Ocular Motility: Evaluation of ductions and versions in all gaze positions.
4. Horizontal Ocular Deviation: Measured at distance and near fixation using the alternate cover test and recorded in prism diopters (PD).
5. Binocular Vision: Assessment of stereopsis using the TNO stereotest.
6. Diplopia Assessment: Documentation of the presence and extent of diplopia, as well as the minimum prism correction required to eliminate it.
7. Ancillary Testing: Analytical and neurological evaluations were performed to exclude associated or underlying pathologies.

### 2.3. Treatment and Outcome Evaluation

Patients were classified according to treatment modality: those managed conservatively with prisms incorporated into their optical correction and those undergoing surgical intervention via lateral rectus muscle strengthening. Surgical treatment was indicated for patients who failed to achieve satisfactory clinical stability or symptom control with prismatic correction, either due to persistent diplopia or inadequate ocular alignment. These patients therefore required surgical intervention to restore functional binocular vision.

A successful sensory outcome was defined as the absence of diplopia following treatment, in association with a successful motor outcome, characterized by residual ocular misalignment ≤5 PD measured at distance fixation on the alternate cover test and stereopsis of 480 arcseconds or better.

### 2.4. Statistical Analysis

Data were analyzed using IBM SPSS Statistics (version 28). Continuous variables were reported as mean ± SD and categorical variables as frequencies and percentages. Normality was assessed using the Shapiro–Wilk test.

Differences between distance and near deviation values were evaluated with a paired Student’s *t*-test (*p* < 0.05). A simple linear regression was performed to analyze the association between preoperative deviation (PD) and lateral rectus resection (mm), and model fit was assessed with R^2^ and residual analysis. A multiple linear regression evaluated the predictive value of distance and near deviation and diplopia frequency for resection amount. A binary logistic regression identified potential predictors of postoperative residual deviation, including age, sex, refractive error, and preoperative distance deviation. Results were presented as odds ratios (OR) with 95% confidence intervals. A significant level of *p* < 0.05 was used for all tests.

## 3. Results

A total of 30 patients (14 men and 16 women), all of Caucasian ethnicity, were included in the study. All were diagnosed with acquired adult esotropia that could not be classified into any of the three traditional subtypes. Therefore, this cohort represents an independent group, possibly associated with new behavioral patterns related to electronic device use. Table 1 summarizes the descriptive characteristics of the esotropia group.

**Figure 1:**
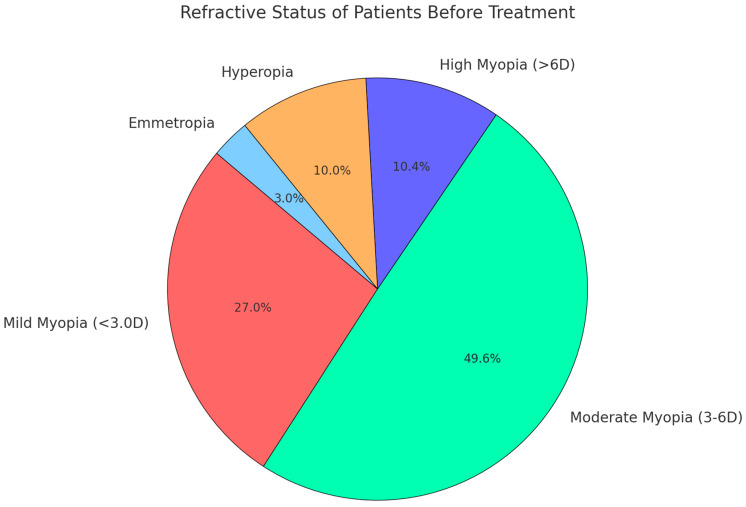
Distribution of the refractive status of patients before treatment. The chart illustrates the percentage of patients classified into different refractive categories: emmetropia (3.0%), hyperopia (10.0%), mild myopia (<3.0 D) (27.0%), moderate myopia (3–6 D) (49.6%), and high myopia (>6 D) (10.4%).

The mean age at presentation for diplopia was 38.13 ± 14.95 years (range: 14–62 years). The mean interval between symptom onset (ocular deviation and/or diplopia) and surgical intervention was 22.52 ± 29.47 months (range: 0.7–92.41 months). Regarding refractive status prior to treatment, 87% of patients were myopic, distributed as follows: Mild myopia (<−3.00 D): 27%, Moderate myopia (−3.00 D to −6.00 D): 49.6%, High myopia (>−6.00 D): 10.4%. Additionally, 10% of patients were hyperopic, and 3% were emmetropic (Figure 1).

Regarding the optical correction, the mean spherical equivalent was −3.19 ± 2.83 D (range: −11.37 to +2.62 D), and the mean cylindrical component was −0.77 ± 0.85 D (range: −3.50 to 0.00 D).

The best-corrected visual acuity (BCVA) was generally good, with a mean value of 1.01 ± 0.09 (range: 0.7–1.2, decimal scale).

**Figure 2:**
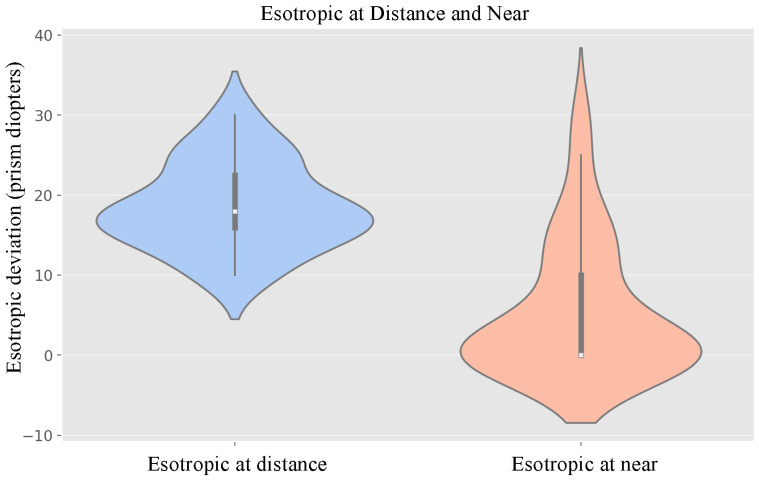
Violin plots illustrating the distribution of esotropic deviation (in prism diopters) under distance (**left**) and near (**right**) fixation conditions. The central lines represent the median and interquartile range, while the overall contour depicts the probability density and distribution of the data.

During the pre-surgical examination, all patients demonstrated acquired esotropia ranging from 10 to 30 prism diopters (PD). The mean preoperative horizontal deviation measured 18.58 ± 5.45 PD at distance fixation and 5.48 ± 8.35 PD at near fixation, with a statistically significant difference between both measurements (*p* < 0.001) (Figure 2). All patients (100%) reported diplopia at distance fixation: occasional in 79.3% of cases and constant in 20.7%.

### 3.1. Etiology and Treatment

With regard to the possible etiology, none of the cases corresponded to the classic subtypes of acquired adult esotropia. However, all patients (100%) reported prolonged use of electronic devices (tablet and/or smartphone) for more than six hours per day.

Comprehensive neurological evaluations and magnetic resonance imaging (MRI) studies were performed in all patients, with no structural abnormalities detected.

Treatment was determined according to the magnitude of the deviation angle:

Conservative management with prisms was employed in cases with deviations ≤10–12 prism diopters (PD).

Surgical treatment was indicated for deviations >10–12 PD.

Overall, 80% of patients (*n* = 24) underwent lateral rectus muscle resection, while the remaining 20% (*n* = 6) were successfully managed with base-out prisms incorporated into their optical correction. All surgical procedures were performed under general anesthesia.

### 3.2. Surgical Findings and Statistical Analysis

In this Results section, 26 of the 30 patients with a follow-up longer than one year were analyzed. Among the patients who required surgical intervention, the mean lateral rectus muscle resection was 4.82 ± 1.23 mm (range: 3.0–7.5 mm).

**Figure 3:**
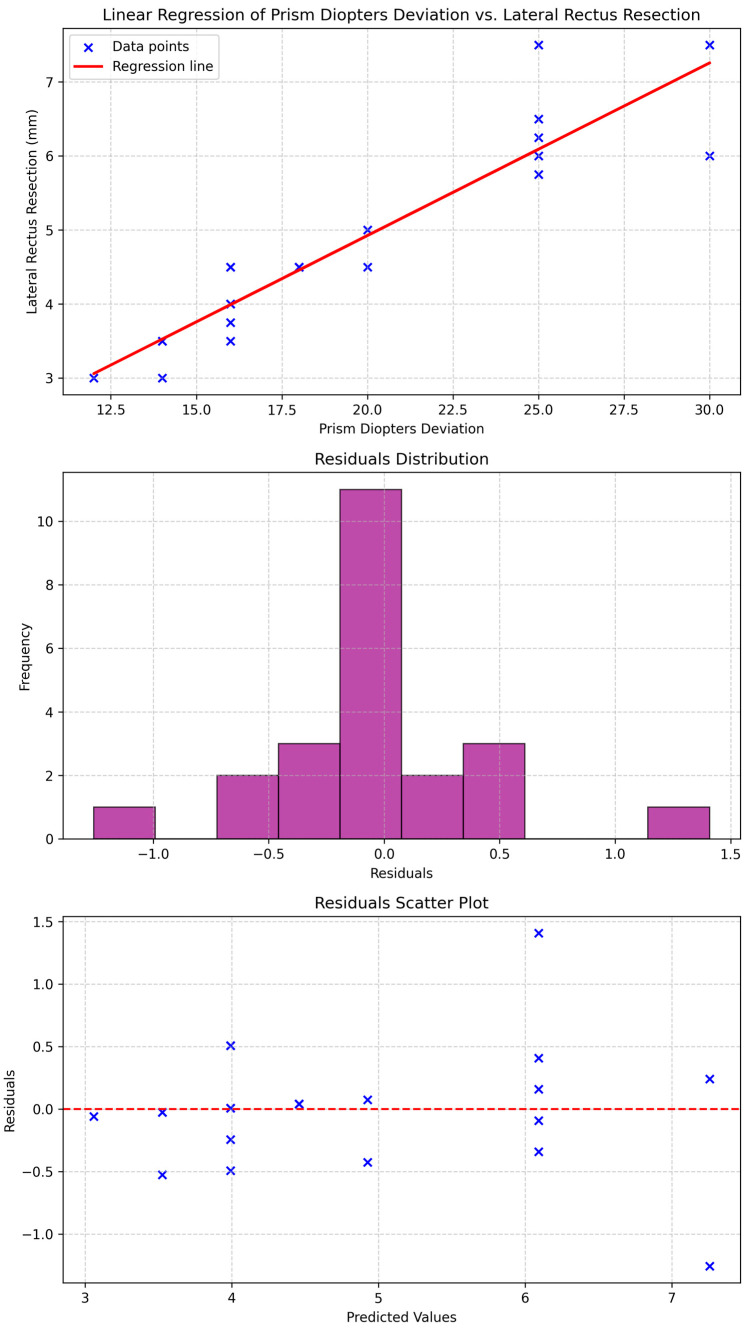
Linear regression analysis of prism diopter deviation versus lateral rectus muscle resection. The upper panel illustrates the regression line with individual data points; the middle panel shows the distribution of residuals; and the lower panel presents residuals plotted against predicted values, confirming the adequacy and goodness of fit of the model.

Analysis of the relationship between the horizontal deviation (in prism diopters, PD) and the extent of lateral rectus resection revealed a strong positive correlation. A linear regression model demonstrated a significant association (*p* < 0.001), in which the preoperative deviation accounted for 86.2% of the variance in resection magnitude (R^2^ = 0.862) (Figure 3). The resulting regression equation was:

Resection (mm) = 0.23 × Deviation (PD) + 0.26

This finding should be interpreted as an exploratory observation derived from the present cohort.

The residuals histogram and scatter plot indicated a well-distributed error pattern centered around zero, suggesting a robust model fit with minimal bias. These findings highlight the clinical utility of preoperative prism diopter deviation as a predictive parameter for determining the appropriate amount of lateral rectus resection.

A multiple linear regression analysis was also performed to evaluate the influence of preoperative deviation at distance and near fixation as well as diplopia frequency (constant or intermittent) on the extent of resection. The results identified distance deviation as the primary predictive variable, with a standardized partial coefficient of 0.934. In contrast, near deviation and diplopia frequency exhibited negligible predictive power, with standardized coefficients of 0.001 and 0.025, respectively (*p* > 0.05), confirming their lack of statistical significance.

Collectively, these findings indicate that preoperative distance deviation is the key determinant for estimating the required degree of lateral rectus resection in patients with acquired adult esotropia.

A binary logistic regression analysis was performed to evaluate the influence of potential predictors on the likelihood of residual deviation following strabismus surgery. The independent variables included age, sex, mean spherical equivalent refraction (Rx) of both eyes, and preoperative distance esotropia. The dependent variable was postoperative alignment outcome, coded as 0 for orthodeviation (reference category) and 1 for residual deviation.

The model demonstrated a moderate fit, with a pseudo R^2^ of 0.485 (*p* < 0.01). The odds ratios (ORs) and 95% confidence intervals (CIs) for each predictor were as follows:

Age: OR = 0.89 (95% CI: 0.77–1.02), suggesting that with each additional year of age, the likelihood of residual deviation decreases, indicating a potential protective effect of age. Sex: OR = 0.35 (95% CI: 0.01–10.28), suggesting that male patients may have a lower, though not statistically robust, likelihood of residual deviation compared to females.

Refraction (Rx): OR = 1.79 (95% CI: 0.80–4.00), indicating a trend toward greater odds of residual deviation with increasing refractive error, although the wide confidence interval suggests variability and lack of statistical significance.

Pre-surgical distance esotropia: OR = 0.68 (95% CI: 0.39–1.19), implying a potential, non-significant protective effect against residual deviation.

As all confidence intervals included the value of 1, none of the analyzed variables demonstrated a statistically significant association with the occurrence of residual deviation. This suggests that the observed relationships may be attributable to random variation, and that age, sex, refraction, and preoperative deviation do not significantly predict postoperative misalignment in this cohort.

### 3.3. Clinical Outcomes

Postoperatively, 65% (*n* = 13) of the surgically treated patients achieved orthodeviation at both distance and near fixation. The remaining 35% (*n* = 7) exhibited residual esodeviation. Among these, 28% (*n* = 2) demonstrated a minimal deviation (1–2°), while the remaining 72% (*n* = 5) presented with residual distance esotropia ranging between 6 and 10 PD. No cases of postoperative near overcorrection or secondary exotropia were observed in this cohort during the follow-up period.

Diplopia resolved in 100% of cases following surgery; however, one patient experienced postoperative regression during the follow-up period and required a second surgical intervention. When sensory surgical success was defined as the complete elimination of diplopia, all patients met this criterion. Motor surgical success, defined as restoration of ocular parallelism in distance fixation, was achieved in 75% (*n* = 15) of patients. The remaining 25% (*n* = 5) showed a marked reduction in their preoperative deviation, with mild undercorrection (residual esotropia of 6–10 PD). Only one patient required reoperation.

No intraoperative complications were reported in any case. The mean postoperative follow-up period was 40.59 ± 20.82 months (range: 12–80 months); only four patients had a follow-up shorter than 12 months and were excluded from the present analysis.

No significant influence of demographic characteristics on postoperative outcomes was observed. Nevertheless, patients were advised to maintain appropriate visual hygiene, particularly limiting excessive use of electronic devices during the postoperative period and in daily visual activities.

## 4. Discussion

The prevalence of acquired adult esotropia has increased significantly in recent years, a trend also observed in our institution. This rise has prompted renewed investigation into the etiology and management of this condition. The cases described in our study do not correspond to any of the three classic subtypes of acquired adult esotropia, suggesting the presence of a new, independent clinical group.

Two major questions arise in relation to this disorder: its etiopathogenesis and its optimal surgical management. Establishing a clear causal link between prolonged use of electronic devices and the onset of acquired esotropia remains complex. However, similar to the well-documented increase in childhood myopia associated with lifestyle changes—such as those described by Chen et al. [14]—a parallel trend may be occurring in this pathology. Lee et al. reported that reducing screen time led to an improvement in the angle of deviation [7]. Furthermore, multiple studies have documented an increased incidence of acute acquired esotropia in adults following the COVID-19 pandemic, correlating this rise with excessive and prolonged electronic device use, particularly during nighttime hours [7,8,10,14,15,16,17,18,19,20,21] (Table 2).

**Figure 4:**
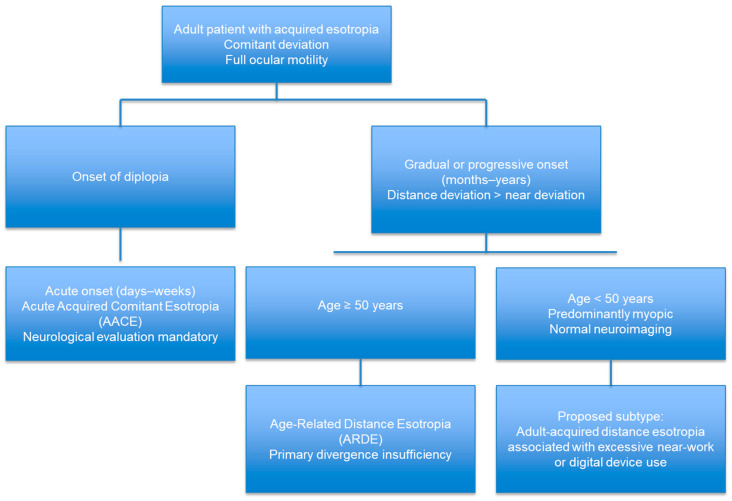
Diagnostic Flowchart for Adult-Acquired Esotropia.

From an etiopathogenic standpoint, excessive near work is believed to increase medial rectus muscle tone, leading to functional shortening and divergence insufficiency secondary to reduced fusional divergence amplitude. In some cases, over-contraction of the ciliary muscle may induce a myopic shift; however, this finding appears to vary according to age and ciliary muscle condition [3]. In Figure 4, a diagnostic flowchart is presented summarizing the proposed classification of adult-acquired esotropia. The flowchart outlines the key clinical features used to differentiate the proposed subtype from other previously described entities, including acute acquired comitant esotropia (AACE), divergence insufficiency, and age-related distance esotropia (ARDE).

In our series, nearly all patients reported excessive use of electronic devices. A practical limitation was that most required these devices for occupational purposes, preventing full discontinuation. Nevertheless, patients were instructed to reduce non-essential screen time, although this alone did not result in complete symptom resolution in any case without either prismatic or surgical treatment.

Intracranial pathology rarely produces this type of esotropia [10,13], but because of its potential severity, neuroimaging and neurological assessment remain essential to rule out central causes. Our findings are consistent with those of Chen et al., who also reported a low incidence of associated neurological disorders [14]. In our cohort, no such cases were identified, since patients with a history of esophoria, microstrabismus, or ocular pathology were excluded from the study.

Regarding age distribution, acquired adult esotropia is typically observed in young to middle-aged adults, consistent with prior reports [8,14,15]. In our cohort, 86% were myopic, with only 12% highly myopic, 9% hyperopic, and 6% emmetropic, which aligns with findings by Zhu et al. [19]. No statistically significant association was observed between refractive error and magnitude of deviation or response to surgical correction. These observations support our view that this condition represents a distinct subtype of acquired adult esotropia that should not be classified within the traditional categories, given its unique clinical characteristics and consistent therapeutic response.

Another possible contributing factor discussed in the literature is divergence insufficiency, classically associated with adults over 60 years of age. However, recent studies have documented similar findings in younger adults [23], suggesting a shifting epidemiologic pattern. In contrast to those reports, where the condition was mostly reversible, our cases did not demonstrate spontaneous resolution, further supporting the hypothesis of a distinct clinical entity.

The second major consideration concerns surgical management. The principal contribution of our study is the proposal of a new treatment indication for acquired adult esotropia. In our cohort, 16% of patients with small-angle deviations (≤10–12 PD) were successfully managed with base-out prisms, consistent with the approaches reported by Wu [11] and Chang [12], among others. The remaining 84% of patients, with deviations >10 PD, required surgical correction, most commonly via lateral rectus muscle resection.

Traditionally, most authors have recommended bilateral medial rectus recession as the primary surgical approach for acquired adult esotropia. However, based on our analysis of the etiopathogenesis and clinical presentation of the cases treated in our institution, we adopted an alternative technique—bilateral lateral rectus reinforcement (resection). This approach directly addresses the distance-related diplopia characteristic of this form of strabismus, enhancing the divergence function of the lateral rectus muscles. Since the deviation predominantly affects distance fixation, targeting the lateral recti is both physiologically and clinically justified.

Our outcomes were highly satisfactory in both motor and sensory terms. Approximately 70% of patients achieved orthodeviation at both distance and near fixation, and among those with residual esodeviation, none exceeded 10 prism diopters. Diplopia resolved in 100% of cases, with all patients maintaining or recovering stereoscopic vision.

In summary, our findings suggest the emergence of a new subtype of acquired adult esotropia, whose etiology remains to be fully elucidated but appears to be strongly associated with excessive use of electronic devices. The proposed lateral rectus strengthening procedure represents an effective surgical alternative, providing excellent functional and anatomical results in this group of patients.

The main limitations of our study include its retrospective design and the limited sample size. However, given the rarity of this pathology and the strict inclusion and exclusion criteria applied, the data remain clinically meaningful. Additionally, a key limitation of this study is that the proposed surgical regression model was derived from a relatively small, single-center cohort. Therefore, the regression should be regarded as preliminary, and its generalizability is limited. External validation in larger, multicenter series is required before this relationship can be reliably applied in broader clinical settings. Future research with larger, prospective cohorts is needed to clarify the pathophysiological mechanisms, determine the true impact of prolonged digital device use, and further evaluate the long-term outcomes and prognosis of this emerging form of acquired comitant esotropia.

## 5. Conclusions

In summary, our findings highlight the following six key clinical features of acquired adult esotropia:

Low-angle esotropia with an insidious onset.

Presence of horizontal diplopia limited to distance fixation, rarely occurring at near.

Wide fusional amplitudes, indicating preserved binocular potential.

Occurrence primarily in emmetropic, myopic (<7–8 D), or mildly hyperopic patients.

Absence of neurological or systemic abnormalities on clinical and analytical evaluation.

Excellent response to both prismatic correction and surgical treatment.

## Figures and Tables

**Table 1:** Descriptive characteristics of the esotropia cohort.

Parameter	Value
Age (years)	38.13 ± 14.95 [15–62]
Sex	Male: 43.33% (*n* = 13); Female: 56.67% (*n* = 17)
Spherical equivalent (D)	−3.19 ± 2.83 [−11.37 to +2.62]
Cylinder (D)	−0.77 ± 0.85 [−3.50 to 0.00]
Refractive status	Myopia: 87% (mild < 3.0 D: 31%; moderate 3–6 D: 57%; high > 6 D: 12%); Hyperopia: 10%; Emmetropia: 3%
Best-corrected distance visual acuity (decimal)	1.01 ± 0.09 [0.7–1.2]
Preoperative examination	Acquired esotropia of 10–30 prism diopters
Treatment	Surgery: 80% (*n* = 24); Prisms: 20% (*n* = 6)
Surgical technique	Lateral rectus muscle resection in all cases (3.5–7.5 mm)
Reoperation rate	3% (1 patient)
Postoperative outcomes	Orthotropia at distance and near: 67% (*n* = 16); Residual esodeviation: 33% (*n* = 8)

**Table 2:** Summary of published studies evaluating near-work and digital device exposure in acquired esotropia.

Study (Year)	No. of Patients	Age (Years)	Exposure to Electronic Devices/Near Work	Intervention/Management
Lee et al., 2016 [7]	12	27.75 ± 11.47	>4 h/day smartphone use for ≥4 months	Device restriction; surgery in residual cases (bilateral medial rectus recession)
Song et al., 2018 [22]	13	22.7 ± 9.7	Excessive smartphone use (>4 h/day)	Conservative (prisms, observation); surgery if persistent (bilateral medial rectus recession)
Topcu Yilmaz et al., 2020 [8]	27	17.8 ± 10.3	≥4 h/day digital screen use (78% of patients)	Prisms or surgery
Mohan et al., 2021 [23]	8	12.5 ± 4.2	The mean duration of smartphone use was 4.6 ± 0.7 h per day	Observational study
Neena et al., 2022 [4]	15	16.8 ± 5.6	>8 h of near activity a day with a mean duration of 8.6 h per day	Prisms and/or surgery (medial rectus recession + lateral rectus resection)
Zhu et al., 2023 [19]	62 (cases)/73 (controls)	25.3 ± 8.5	≥8 h/day near-work (56.5% of cases); frequent nocturnal device use	Mixed (prisms, botulinum toxin, surgery)
Present study	30	38.13 ± 14.95	>6 h/day electronic device use in 100% of patients	Prisms or Surgery (lateral rectus resection)

## Data Availability

The data presented in this study are available on request from the corresponding author.
